# Timing and Nutrient Type of Isocaloric Snacks Impacted Postprandial Glycemic and Insulinemic Responses of the Subsequent Meal in Healthy Subjects

**DOI:** 10.3390/nu16040535

**Published:** 2024-02-14

**Authors:** Xinling Lou, Zhihong Fan, Jinjie Wei, Xiyihe Peng, Jiahui Hu, Xuejiao Lu, Anshu Liu

**Affiliations:** 1 College of Food Science and Nutritional Engineering, China Agricultural University, Beijing 100083, China; xinlinglou@cau.edu.cn (X.L.); 13312551374@163.com (J.W.); pxyh0831@cau.edu.cn (X.P.); hujiahui1023@126.com (J.H.); feirlu@163.com (X.L.); liuanshu@cau.edu.cn (A.L.); 2 Key Laboratory of Precision Nutrition and Food Quality, Department of Nutrition and Health, China Agricultural University, Beijing 100083, China

**Keywords:** chicken breast, apple, macadamia nut, glucose excursion, snack timing

## Abstract

The aim of the study was to explore the impact of both the macronutrient composition and snacking timing on the postprandial glycemic insulinemic responses and food intake. Seventeen healthy female volunteers completed the randomized crossover trials. The volunteers were provided a standard breakfast and lunch at 8:00 and 13:00, respectively, and an ad libitum dinner at 18:00. Provided at either 10:30 (midmorning) or 12:30 (preload), the glycemic effects of the three types of 70 kcal snacks, including chicken breast (mid-C and pre-C), apple (mid-A and pre-A), and macadamia nut (mid-M and pre-M), were compared with the non-snack control (CON), evaluated by continuous glucose monitoring (CGM). The mid-M showed increased insulin resistance after lunch compared with CON, while the pre-M did not. The pre-A stabilized the glycemic response in terms of all variability parameters after lunch, while the mid-A had no significant effect on postprandial glucose control. Both the mid-C and pre-C improved the total area under the glucose curve, all glycemic variability parameters, and the insulin resistance within 2 h after lunch compared with CON. The pre-C attained the lowest energy intake at dinner, while the mid-A and the mid-M resulted in the highest. In conclusion, the chicken breast snack effectively stabilized postprandial glycemic excursion and reduced insulin resistance while the macadamia snack did not, regardless of ingestion time. Only as a preload could the apple snack mitigate the glucose response after the subsequent meal.

## 1. Introduction

Snacking is a habitual behavior in most people. It is reported that 73% of insulin-treated diabetics consume snacks and 87% of them enjoy snacking [1], as they are prone to have hypoglycemia before meals. Increased snacking frequency of low-nutrient-density food has been shown to be associated with an elevated risk of obesity and chronic disease [2]. However, wise choices of snacks can not only improve the nutrition quality of the daily diet, but also promote satiety throughout the day [3,4].

A growing body of evidence indicates that the health consequences of snacking may depend on the time of consumption and the nutrient composition. A previous study showed that consuming snacks between meals was helpful for curbing the rise in glucose levels after the subsequent meal [5]. Imai et al. [6] and Nitta et al. [7] reported that eating a mid-afternoon snack could suppress glucose excursions, while snacking post-lunch or post-dinner could not. Moreover, consuming snacks 2 h before dinner resulted milder postprandial blood glucose rises than 4 h before dinner [8], which suggested that the timing of ingestion could be a key factor in the physiological effect of snacking.

Snacks can be ingested after meals, right between two meals, or as a preload food at 30 min prior to a meal. Preloading foods rich in protein (such as whey protein, fish, and beef), carbohydrate (such as fruits), or fat (such as olive oil, margarine, and almonds) has been reported to be able to attenuate postprandial blood glucose response [9,10,11] in recent years. Eating protein-rich food between or before meals [7,12,13] may elicit a second meal effect, and the glycemic benefits of protein snacks may be attributed to delayed gastric emptying, enhanced glucose-stimulated insulin release, and decreased insulin clearance [10]. Masutomi et al. [8] studied the glycemic effects of four popular snack foods on blood glucose at the next meal. They found that the effects of the snacks were associated with their macronutrient compositions, and the carbohydrates and soluble fiber in snacks were key contributors to the glycemic stabilizing effect.

It has been noticed that most of the previous studies investigated the glycemic effect of snacks on dinner, while the possible effect of snacks on lunch remains unclear. In addition, a comparison of the glycemic and insulinemic effects of snack timing focused on the difference in macronutrient compositions has not yet been reported. Research on circadian rhythm has discovered that, compared with food intake in the late part of the day, food consumed earlier is more beneficial for the prevention of obesity and chronic diseases such as diabetes [14]. As a considerable percentage of people do not take in enough energy and protein in their breakfast, snacks in the morning can make up for the nutrient supply before lunch.

Immoderate snacking may increase the risk of obesity and anxiety, possibly due to excess energy intake [15]. Therefore, the glycemic regulation effect of snacks should be achieved at the expense of no or minimized energy surplus and discussed on the basis of calorie intake.

In the present study, we chose chicken breast, apple, and macadamia nut to represent high-protein, high-carbohydrate, and high-fat snacks, respectively. The macadamia nut is characterized by a fat content of over 70% and a protein content less than 10%, while the flavored chicken breast is high in protein and low in fat. In contrast, the apple is a carbohydrate-dominant food which is low in both protein and fat. The energy intake from each snack was set as 70 kcal, and the timing of the snack was either midmorning or pre-lunch. We hypothesized that both the macronutrient composition and the timing of snacking would make a difference in terms of post-lunch glycemic insulinemic regulation efficiency and food intake at dinner. Based on the previous report, we also assumed that as a carbohydrate snack, the preload apple might curb postprandial glucose more effectively than the mid-morning apple.

## 2. Materials and Methods

### 2.1. Participants and Ethics

A total of 17 female students volunteered via social media advertisements and were included if the following criteria were satisfied: no record of any metabolic disease; body mass index (BMI) between 18.5 and 24.9 kg/m^2^; regular sleep–wake cycle; consuming 3 meals a day regularly; and having a regular menstrual cycle. Participants were excluded from the study if they were allergic to any food used in the study; had been on a weight loss diet or any special diet within the past 6 months; had eating disorders; had digestive disorders or frequent gastrointestinal discomfort; had the habits of smoking, drinking alcoholic beverages, or drinking coffee; or were taking any medications or supplements known to affect metabolism.

The basal metabolism rate (BMR) was measured using the body fat scale (HBF-371, OMRON, Yangzhou, China), and the fat mass and visceral fat indices were obtained in the meantime. Resting blood pressure was measured in duplicate with an electronic blood pressure monitor (HEM-7200, OMRON, Dalian, China) and the waist: hip ratio was measured with tape. All eligible individuals who passed oral glucose tolerance tests (OGTT) (fasting glucose < 6.1 mmol/L, peak glucose < 10.0 mmol/L and 2 h glucose < 7.8 mmol/L) signed written informed consent forms.

The study protocol was conducted in full compliance with the Helsinki Declaration, granted by the Ethics Committee of China Agricultural University (protocol code CAUHR-20230301 and date of approval was 6 March 2023), and registered on the Chinese Clinical Trial Registry (ChiCTR2300070200).

### 2.2. Study Design and Procedures

The study consisted of seven separate experimental conditions in a randomized, open-label, crossover design with each session being separated by a wash-out period of at least one day. An online computer software program (http://www.randomizer.org; accessed on 1 March 2021) was used for the simple randomization of the sequence of the treatments [16]. If any subject was menstruating during the trial, the schedule was rearranged. In addition, participants were told to avoid strenuous exercise throughout the trial.

The subjects wore continuous glucose monitors (CGM) (Abbott, Shanghai, China) on the first day of the study, and an over-1-day observation period was set for glucose monitoring stabilization [17]. Moreover, smart bracelets (Xiaomi, Beijing, China) were used to monitor the physical activity and sleep during the trial. On the day before the first test session (Day 0), participants were instructed to consume breakfast, lunch, and dinner in university canteen at 8:00, 12:00, and 18:00, respectively, and abstain from coffee, tea, alcohol, and any dietary supplement. On the test day (Day 1), the subjects were given breakfast at 8:00 and lunch at 13:00. An isocaloric snack of any one of three choices, including (1) flavored chicken breast (C), (2) fresh unpeeled apple (A), and (3) macadamia nut (M), was given at either 10:30 (mid-morning snack) or 12:30 (preload prior to lunch). The three choices represented protein-, carbohydrate- and fat-dominant snacks, respectively.

**Figure 1:**
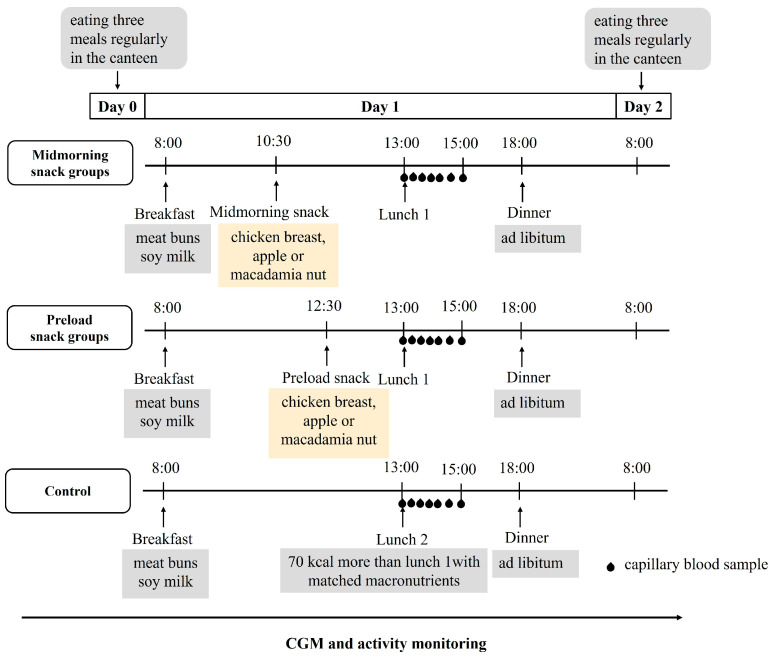
Summary of the study’s experimental design. CGM, continuous glucose monitor. All participants underwent seven trial conditions in a randomized order, including chicken breast as mid-morning snack (mid-C); chicken breast as preload snack (pre-C); apple as mid-morning snack (mid-A); apple as preload snack (pre-A); macadamia nut as mid-morning snack (mid-M); macadamia nut as preload snack (pre-M); and no snack as control (CON).

According to our previous study, a 70 kcal apple preload could significantly curb the postprandial glycemic excursion of the subsequent hyperglycemic response meal [9]. Thus, we set the energy counts of all three snacks as 70 kcal (Table 1). At 18:00, the subjects had their dinners ad libitum, and the amount of food they consumed was recorded. Participants were required to consume all the food in the research unit, including snacks and three meals. In addition, they were required to minimize physical activity as much as possible after breakfast and remain seated in the research unit after lunch until the end of the test session (22:00). From the test day to 8:00 the next day (Day 2), participants were not allowed to consume anything other than water. The whole experimental design is shown in Figure 1.

### 2.3. Test Meal Challenge

The standardized breakfast, containing 472 kcal (61% energy from carbohydrates, 24% from fat, and 15% from protein), consisted of two meat buns and a cup of soy milk. In order to ensure that the energy provided before dinner was consistent across all treatments, we provided the subjects with two kinds of lunch meals, which were tightly matched in macronutrient distribution: 60% from carbohydrates, 25% from fat, and 15% from protein (Table 2). The dinner was a mixture of white rice, seasoned seaweed, and egg, freshly made in the laboratory, with a macronutrient distribution as follows: 26% from fat, 60% from carbohydrate, and 14% from protein. The eligible subjects tried all three meals prior to the test sessions to ensure that the food had good acceptability and that they could finish eating within the prescribed time. All the test meals were prepared by the study team on the day of each session, immediately served to the volunteers, and consumed within 15 min to avoid possible retrogradation of starch.

### 2.4. Continuous Glucose Monitoring

The glucose levels of the subjects were monitored using a continuous glucose monitor (CGM). The CGM was inserted one day before the first condition at approximately 8:00, and the sensor was removed 24 h after the last condition. The data reported in this paper represent interstitial glucose levels recorded every 15 min for 14 consecutive days, and occasional missing values were imputed using linear interpolation [18].

### 2.5. Blood Collection and Analysis

At the beginning of each lunch meal (0 min) and 15, 30, 45, 60, 90, and 120 min after meal ingestion, 150 µL capillary blood samples from the fingertip was collected into EDTA K2-treated centrifuge tubes (WanDGL Ltd., Jinan, China). Within 30 min after blood collection, the samples were centrifuged at 1000× *g* for 15 min, and 60 μL of the supernatant plasma was dispensed into 0.5 mL Eppendorf tubes and stored at −80 °C until the analyses. Insulin concentrations were determined using an ELISA-based test kit (MEIMIAN Ltd., Yancheng, China).

### 2.6. Data Processing and Statistical Analysis

The glycemic responses after snacks (10:30~13:00), lunch (13:00~15:00), dinner (18:00~22:00), and 24 h (Day 1 8:00~Day 2 8:00) were expressed as the total areas under the curve (tAUCs), calculated using the trapezoidal approximation method and the absolute values of glucose [19]. Glycemic variability parameters included: mean (Mean), maximum (Max), and minimum (Min) glucose levels; large amplitude of glycemic excursions (LAGE); glucose standard deviation (SD); glucose coefficient of variation (CV); and J-index, calculated as 0.324 × (mean glucose + SD glucose)^2^ [20]. Moreover, the time above range (TAR), time in range (TIR), time below range (TBR), and ΔM_L-D_ as the differences between the postprandial glucose max values after lunch and dinner were also calculated to represent the glucose excursion over a day [21]. In order to improve the detection ability in healthy people, the glucose range was adjusted to the fasting glucose to 2.5 higher than the fasting glucose value.

The postprandial insulin data were based on the percent change of insulin relative to the fasting insulin concentration to eliminate inter-personal variability. The incremental area under the curve (iAUC) of postprandial insulin responses and the index of postprandial insulin resistance (HOMA-PP), defined as iAUC_0–120_ glucose × iAUC_0–120_ insulin/22.5, were calculated [22].

The power calculation was conducted using the PASS 13 Power Analysis and Sample Size software version 21.0.3 (NCSS, Kaysville, UT, USA). According to the study design, a minimum sample size of 14 was required in order to observe a difference in AUC between treatments with an 80% power level and 5% significance level, assuming that the standard deviation (SD) was lower than 3400 min·mg/dL based on a previous study [8]. To allow for a 30% anticipated attrition rate, the number of recruited participants was extended to 18.

All the statistical analysis was performed using SPSS version 25.0 (SPSS Inc., Chicago, IL, USA). A linear mixed-effects model was used to assess the difference between treatments and across time. When significant (*p* < 0.05), post hoc tests were performed using Bonferroni corrections. The variables were presented as the mean ± standard deviation (SD) or the mean value with standard error (SE).

## 3. Results

### 3.1. Baseline Characteristics of Participants

**Figure 2:**
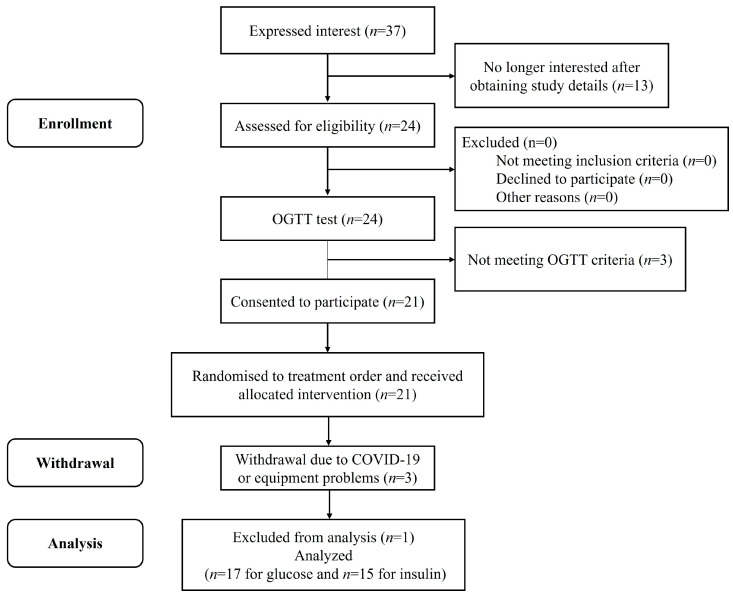
Consolidated standards of reporting trial (CONSORT) flow diagram of the study subjects.

A flow chart representing the study subjects is shown in Figure 2. Eighteen volunteers passed the screening and completed all the test protocol, but one was excluded because of non-adherence to the study protocol. Fifteen participants’ capillary blood was collected for insulin analysis, as the remaining two participants had difficulty in collecting blood samples. All participants’ baseline characteristics are presented in Table 3. None of the participants reported any adverse events during the test sessions.

### 3.2. Postprandial Glycemic Response after Snacks and Lunch

**Figure 3:**
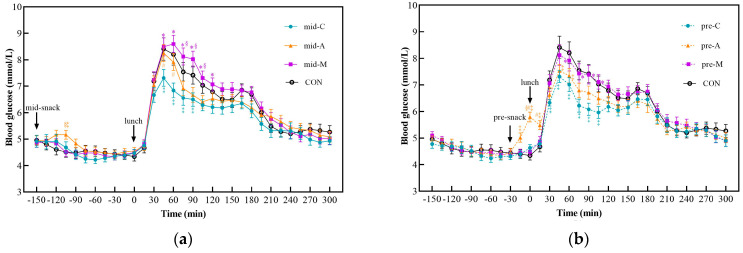
Postprandial glycemic curves from 10:30 to 18:00. Mid-C, chicken breast as mid-morning snack; pre-C, chicken breast as preload snack; mid-A, apple as mid-morning snack; pre-A, apple as preload snack; mid-M, macadamia nut as mid-morning snack; pre-M, macadamia nut as preload snack; CON, no snack as control. Data show the mean ± SE (*n* = 17). * Macadamia nut treatments differ from chicken breast counterparts; ^§^ macadamia nut treatments differ from apple counterparts; ^#^ apple treatments differ from chicken breast counterparts; ^‡^ snack treatments differ from CON treatment (*p* < 0.05). (**a**) Postprandial glycemic curves of midmorning snacks; (**b**) Postprandial glycemic curves of preload snacks.

The postprandial glucose curves and glycemic parameters are shown in Figure 3 and Table 4, respectively. The postprandial glycemic responses of the chicken breast treatments were remarkably lower than the CON, manifesting as a significant lower glucose level from 45 min to 90 min of the mid-C and from 30 min to 105 min of the pre-C, respectively. All the glucose parameters after lunch (Table 4) in the two chicken breast groups were significantly lower than those in the CON. In the apple groups, only the pre-A resulted in a lower glucose level within 45 min and 60 min than the CON did. All glycemic parameters of the pre-A after lunch, except the tAUC, were lower than those of the CON. However, the mid-A, mid-M, and pre-M had no significant differences in any of the items compared with the control group.

Among the three snacks, the macadamia nut groups showed the highest PGR, followed by the apple groups, and the chicken breast groups elicited the lowest. The mid-M led to a significantly higher glucose level from 45 min to 120 min, while the pre-M resulted in a higher value from 60 min to 105 min than the mid-C and pre-C did, respectively. Similarly to the postprandial glucose curve, all parameters of the macadamia nut treatments after lunch were significantly higher than those of their chicken breast counterparts regardless of the ingestion time. Moreover, the mid-M treatment also caused a significant higher glucose level from 75 min to 105 min than the mid-A did.

As for the glycemic parameters after lunch, the macadamia nut treatments elicited significantly higher J-index values than their apple counterparts, regardless of the snack timing. In addition, the mid-M group led to a higher tAUC value from 13:00~15:00 than the apple treatments did. As a preload snack, the LAGE and CV of apple were significantly lower than those of the macadamia nut groups. Compared with chicken breast, only the apple preload could significantly reduce the CV value, while consuming apple between meals resulted in higher glucose levels at 45 min and 60 min than those of the mid-C.

With respect to the different snack timings, neither the chicken breast nor the macadamia nut groups showed significant time differences. However, the pre-A elicited higher glucose values at 0 min and 15 min than the mid-A did (*p* < 0.05). Among the postprandial glycemic parameters after lunch of different timings of snack consumption, only the LAGE and CV values of the mid-A group were significantly higher than those of the pre-A group.

### 3.3. Postprandial Insulinemic Response after Lunch

**Figure 4:**
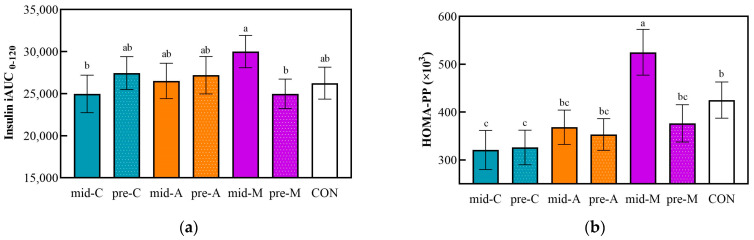
Postprandial capillary insulin iAUC from 13:00 to 15:00 (**a**) and HOMA-PP (**b**). Mid-C, chicken breast as mid-morning snack; pre-C, chicken breast as preload snack; mid-A, apple as mid-morning snack; pre-A, apple as preload snack; mid-M, macadamia nut as mid-morning snack; pre-M, macadamia nut as preload snack; CON, no snack as control. Data are the mean ± SE (*n* = 15). Significant differences (*p* < 0.05) are represented by different letters on the bars.

The iAUC_0–120_ and HOMA-PP based on postprandial capillary insulin and glucose concentrations are shown in Figure 4. The mid-M induced the highest insulin iAUC_0–120_ value, while the results of the mid-C and pre-M were the opposite. In addition, the HOMA-PP of mid-M was 1.6 times and 1.4 times that of the mid-C and mid-A (*p* < 0.05), respectively, and insulin resistance was recovered by the preload treatment (pre-M).

### 3.4. Energy Intake and Postprandial Glycemic Response at Dinner

**Figure 5:**
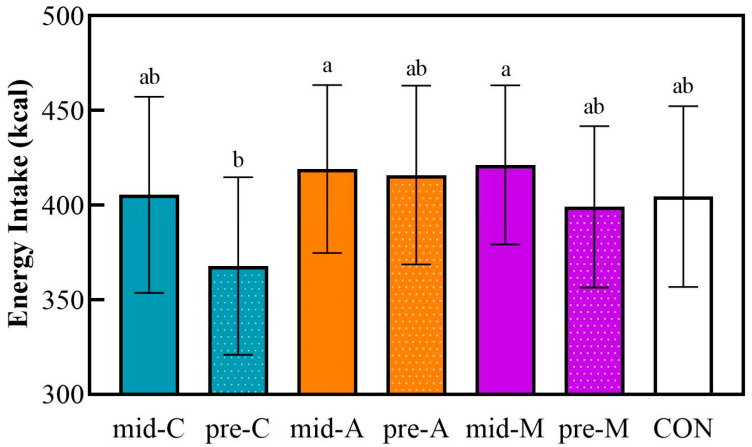
The energy intake at a subsequent macronutrient-balanced dinner. Mid-C, chicken breast as mid-morning snack; pre-C, chicken breast as preload snack; mid-A, apple as mid-morning snack; pre-A, apple as preload snack; mid-M, macadamia nut as mid-morning snack; pre-M, macadamia nut as preload snack; CON, no snack as control. Data are the mean ± SE (*n* = 17). Significant differences (*p* < 0.05) are represented by different letters on the bars.

Figure 5 shows the energy intake after a macronutrient-balanced dinner, the meal subsequent to the test lunch.

The pre-lunch chicken breast induced the lowest energy intake at dinner, while the mid-A and the mid-M led to the highest energy intake at dinner. However, the food consumption at dinner showed no significant differences based on the snack treatments compared with the isocaloric non-preload control. There were no differences in the postprandial glycemic responses or parameters within any of the treatments in spite of the different energy intakes.

### 3.5. 24 h Glucose Trace

**Figure 6:**
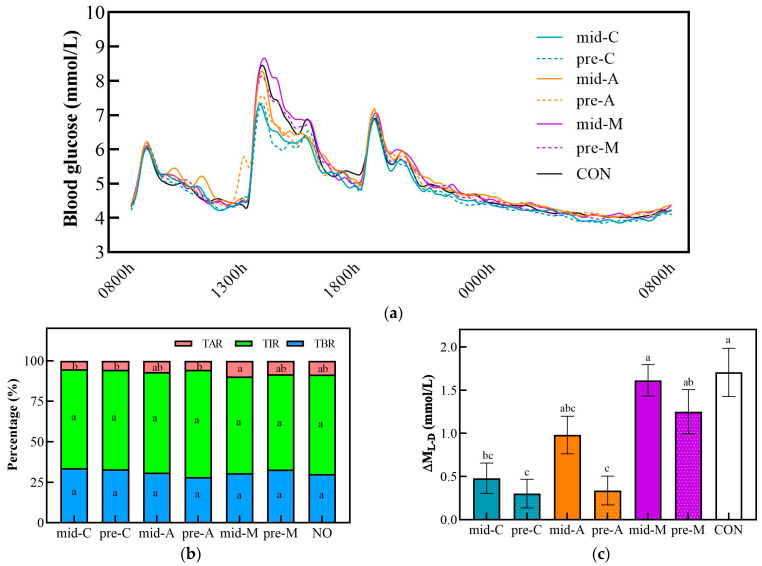
The glucose trace (**a**), percentage of time in different ranges (**b**), and ΔM_L-D_ (**c**) for 24 h in snack testing trial conditions. Mid-C, chicken breast as mid-morning snack; pre-C, chicken breast as preload snack; mid-A, apple as mid-morning snack; pre-A, apple as preload snack; mid-M, macadamia nut as mid-morning snack; pre-M, macadamia nut as preload snack; CON, no snack as control. Data are mean ± SE (*n* = 17). Significant differences (*p* < 0.05) among different groups are represented by different letters.

Figure 6 and Table 5 show the glucose trace and glucose excursion parameters for 24 h in seven test trials. Lunch preloads of chicken breast and apple produced lower ΔM_L-D_, 24 h Max, 24 h LAGE, and 24 h SD values than the CON did. Moreover, compared with the CON group, the mid-C also reduced the ΔM_L-D_, 24 h Max, and 24 h LAGE, while the mid-A, mid-M, and pre-M did not.

Based on the same timing, the three types of snacks showed no significant differences in the 24 h Mean and 24 h tAUC. However, when taken as mid-morning snacks, the macadamia nut caused a higher blood glucose excursion than the chicken breast did, as shown by the higher TAR, 24 h LAGE, and 24 h CV values. In addition, the macadamia nut snack elicited significantly higher ΔM_L-D,_ 24 h Max, and 24 h SD than its chicken breast counterpart did, regardless of the timing. There were no differences in these glycemic parameters between apple snack interventions and their chicken breast counterparts except for the 24 h Max; the mid-A achieved a significant increase compared to the mid-C. Moreover, the pre-A treatment attained the smallest ΔM_L-D_, 24 h SD, and 24 h CV values, which were significantly lower than those of the pre-M. Similarly to previous results, there was no significant difference in any 24 h glucose fluctuation parameter between different snack timings.

## 4. Discussion

The present study found that, based on a 70 kcal isocaloric consumption, the types of macronutrients in before-lunch snacks had a significant impact on their glycemic effects and insulin resistance, although they had little effect on energy intake at dinner. The chicken breast treatments reduced the glycemic response and insulin resistance compared with the non-preload control, while the macadamia nut treatments showed no improvement compared with the control group, irrespective of timings. The timing of snacking, however, only made a difference in the apple group, i.e., for the carbohydrate snack. The pre-lunch apple stabilized the post-lunch blood glucose, but the between-meal apple failed to achieve glycemic improvement.

Snacks are generally recommended to be under 200 kcal for adults [23]. In this study, 70 kcal was set as the energy intake of the snacks based on our previous study, which found that an apple preload of 70 kcal could remarkably improve the blood glucose stability at the subsequent meal [9]. In order to eliminate the differences in energy intake between the snack groups and the control group, the lunch energy of the non-snack control was 70 kcal higher than that of the snack groups, but with exactly the same macronutrient composition.

Fruits and nuts are recommended to be included in the daily diet in many countries [24], as the consumption of fruit and nuts is proven to be inversely associated with the risk of certain cancers, diabetes, and cardio-metabolic diseases [25,26,27,28,29,30]. As a glycemic-friendly food, apple is not only a low-GI food, but can also effectively mitigate the postprandial glycemic response when ingested as a preload food [9]. Moreover, apple is available across seasons and throughout the world. The macadamia nut is the nut with the highest fat content and the lowest protein content [31], but it has been scarcely studied in terms of glycemic effects. Meat-based snacks are also popular in many countries, among which chicken breast is especially well recognized as a high-protein and low-fat food material. Based on the above consideration, we chose apple, macadamia nut, and chicken breast as the three typical snack samples.

The present study found that the chicken breast could best stabilize the postprandial glycemic response after lunch irrespective of timing. Meng et al. [32] found that the macronutrient composition of the pre-meal food impacted the glycemic response and the glucose rise at the next meal, as the high-protein food showed the mildest postprandial glycemic response. It is well established that protein preloads consisting of a wide range of protein types and amounts (about 12–55 g protein) can blunt the glycemic response to a subsequent carbohydrate-rich food or meal [13,33,34,35]. Skalkos et al. [36] found that using lupin biscuits (114 kcal) as mid-meal snacks reduced postprandial glucose levels 90 min after dinner in type 2 diabetes, which may be related to the high protein content of the snack. However, a comparison of snacking in a mid-meal manner (2.5 h before meal) and a preload manner (0.5 h prior to a meal) has not yet been reported. The present study found that, even when eaten 150 min before a meal, the chicken breast exerted an influence on the postprandial glycemic response as much as the preload did.

The minimal glucose excursions achieved by the chicken breast snack may be explained by the second-meal effect of protein. It has been reported that 55 g of whey preload reduced the glycemic response to a mashed potato meal consumed later in type 2 diabetic patients, and the result was attributed to delayed gastric emptying and sustained release of gut-derived signals including glucagon-like peptide-1 (GLP-1), cholecystokinin (CCK), and glucose-dependent insulinotropic polypeptide (GIP) [33]. These incretins rose significantly within 30 min after the ingestion of protein and circulated in the plasma for hours [33,34]. Similarly, an oyster meat (0.2 g/kg) preload reduced post-meal glycemia, with an increase in the GLP-1 concentration [37]. Consistent with the result of the soy milk and dairy milk preload [38], in the present study, the chicken breast snack did not elicit a significant increase in insulin concentration, which might be explained by its limited protein content (13 g).

Compared with the chicken breast groups, the macadamia nut groups showed higher postprandial glycemic responses at both of the timings. None of the nut snacks treatments could reduce the glucose excursion compared with the control. A number of studies proved that, compared with a low or normal fat diet, a high-fat diet leads to an increase in postprandial peak glucose levels at the next meal, which might be due to attenuated insulin sensitivity [39] and lowered carbohydrate utilization [40]. Although lipids evacuate more slowly from the stomach than carbohydrates or proteins do because of their high caloric content [41], the 9.5 g dose of nut in our trial might have been too low to affect the pace of stomach evacuation.

Previous studies on the benefits of nuts have mainly focused on peanuts and almonds. Crouch et al. [42] found that a 14.2 g dry-roasted almond (84 kcal, 0.7 g carbohydrate, 4 g protein, 7.5 g fat) preload could reduce glucose response after a 75 g glucose challenge. Similarly, an almond (20 g) preload before three major meals could suppress the fluctuation of glucose throughout the day [43]. However, our results indicated that the beneficial effect of nuts on the postprandial glycemic response might depend on the type of nut.

The effect of nuts might be associated with several factors: first, the proportion of fat to protein. Peanuts and almonds have a fat: protein ratio of about 2:1, while the macadamia nut’s ratio is about 9:1 [31]. Masutomi et al. [8] reported that, inconsistent with common sense, a black soybean snack (200 kcal) before dinner failed to curb the postprandial glucose excursion after dinner as much as the sweet potato did. One of the reasons might lie in that the black soybean snack was a fried food, which contained even more fat than protein (13.3 g vs. 12.3 g), greatly increasing the ratio of fat: protein and masking the benefits of the protein. Second, the fiber content: Consuming a mixture of fat and fiber before a rice meal helped to reduce the fluctuation of blood glucose [44]. However, the fiber content of macadamia nuts is much lower than that of almonds [31]. Finally, the texture of the food matrix may lead to different glycemic and insulin response [45]. The lower chewiness of the macadamia nut compared with that of the almond might have raised the digestion rate.

In this study, when used as a mid-meal snack, the macadamia nut resulted in the highest glucose response, postprandial insulin secretion, and insulin resistance, while the macadamia nut preload improved the above two insulin parameters. Gentilcore et al. [46] assessed the effects of 30 mL water or 30 mL olive oil 30 min before a mashed potato meal and found that the oil preload led to lower insulin levels only within two hours after the meal, while the opposite was true later on. In that study, the oil preload increased postprandial GLP-1 within only 150 min after the meal. Therefore, different physiological effects might be explained by different timings of fat intake.

In the present study, we focused on the changes in glucose two hours after lunch, as 70 kcal of apple (137 g) with 15 g sugar did not increase the tAUC within 5 h after lunch despite the slightly increased amount of carbohydrates. Previous studies [9,47,48,49] have confirmed that ingesting fruits as a preload instead of co-ingestion or after-meal dessert could facilitate postprandial glycemic management. However, the effect of eating a moderate amount of fruit as a between-meal snack has been tested previously. The present study showed that, when ingested right between meals, apple failed to provide any benefit to postprandial glycemic stability. This result suggests that the timing of carbohydrate snacks might be an important factor in terms of their glycemic impact.

The GLP-1 is not likely to be an explanation of the benefits of an apple preload, as fruit preloads did not significantly increase the secretion of GLP-1 [47,48]. Previous studies have suggested that the glycemic benefits of an apple preload might be attributed to the early-phase insulin response elicited by the sugar moiety [49,50] and the catalytic effect of a small amount of fructose on liver glucose metabolism [51,52]. A small rise in insulin induced by the apple snack hardly lasted 150 min and did not impact the following meal. As our results indicate, consuming apple between meals led to no significant differences in glucose or insulin levels compared with the control group at the beginning of lunch. Mishra et al. [53] did find that eating kiwifruit at either 90 min or 30 min before a meal could reduce the postprandial glucose response. However, it is worth noting that in that experiment, kiwifruit replaced half of the energy of the meal, while in our experiment, the energy provided by the apple was only about 10% of that of the lunch. A larger amount of carbohydrates might elicit longer-lasting insulinemic impact.

The non-sugar ingredients in apple, such as polyphenols and dietary fiber, might also have effects on blood glucose control. The polyphenols in fruits could inhibit the activity of α-glucosidase, retard the process of digestion, and improve insulin sensitivity [54,55,56]. Dietary fiber, such as pectin, in apple may delay gastric emptying and slow down glucose absorption via increasing the viscosity of the intestinal contents [57,58]. However, when ingested in mid-morning, most of the effects of polyphenols and fiber on gastrointestinal processes diminished before lunch [53]. Therefore, a mid-morning apple snack failed to affect the post-lunch blood glucose level.

It was noticed that the pre-meal chicken breast induced the lowest energy intake at dinner, which might be the combined result of the strong satiety resulting from protein and the timing. It is well established that foods with high protein are more satiating than the isoenergetic ingestion of carbohydrate or fat [59]. The alanine-rich feature of chicken breast might be a plus for its protein-induced satiety [60]. It is reported that, compared with dividing a large portion of food into five snacks, ingesting that large portion of food at one time could more effectively raise the level of GLP-1 and suppress the feeling of hunger [61]. In the present study, because of the short interval between the preload snack and the meal, the preload food might have been perceived as a part of the lunch meal, thus eliciting enhanced satiety and reduced energy intake at dinner.

Though nuts are regarded as a highly satiating food type and nut consumption has been reported to be associated with decreased appetite [62], in our study, the mid-morning macadamia snack resulted in the highest energy intake at dinner. On one hand, nut intake may not always help to reduce hunger and enhance GLP-1 levels [63]; on the other hand, the nut portion in the present study might have been too small to make a difference.

In spite of the effects achieved herein of glycemic stabilizing and reduction in food intake, the chicken breast snack was shown to elevate the meat intake and protein energy throughout the day. Being a white meat, skin-less chicken meat is preferred as a replacement for red meat as its consumption is not associated with all-cause mortality [64]. Considering the benefits of adequate protein in terms of preventing excess loss of muscle [65], a supplement of 12.9 g protein from chicken breast to a lunch containing 23.3 g protein sounds acceptable.

To the best of our knowledge, this was the first trial to compare the effects of snacks on blood glucose variation, insulinemic response and energy intake at 30 min pre-meal or right between breakfast and lunch. The three snacks used in this study were natural foods that best represented protein-, carbohydrate- and fat-dominant macronutrient compositions, respectively. In addition, the comparison between the control group and the snack groups was based on isocaloric intake, which is a point that has not been considered in most previous studies. The total energy among the test groups was strictly controlled to rule out the possibility that the postprandial glycemic response and appetite were affected by extra energy intake from the snacks. Finally, the participants were instructed to consume three meals a day at fixed times prior to all test days to rule out any possible confounders caused by meal time differences.

However, the limitations of our study must be considered. Firstly, because only healthy young women were recruited for the study in order to limit heterogeneity, the results of this study may not apply to other groups of people. Secondly, the long-term outcomes of snacking patterns cannot be simply extrapolated from the results of this acute trial. Finally, the secretion of incretin hormones was not investigated in the present study.

## 5. Conclusions

Given the fact that eating snacks is a common behavior in most societies, it is relevant to optimize the snacking choices and snacking pattern for possible glycemic benefits without extra insulin secretion or energy intake.

In conclusion, the present study found that the high-protein and low-fat chicken breast snack, but not the high-fat and low-protein macadamia nut, could effectively curb the glycemic excursion while reducing insulin resistance after a meal compared with the non-snack control, irrespective of ingestion timing. However, the timing of ingestion seemed to be a determinant of the glycemic effect of the carbohydrate snack, as only the pre-lunch apple mitigated the post-lunch blood glucose response without increasing the insulin response and insulin resistance. The wise use of snacks, especially preload snacks, may provide a simple approach to achieving a milder postprandial glycemic excursion without the costs of extra insulin load or energy surplus. The result of this trial should be further verified in other groups, such as prediabetics, diabetics, children, adolescents, and male adults, as well as among those with various meal patterns and dietary patterns, for they may have different habits of consuming snacks and might have different physiological responses to the snacks. Moreover, the physiological mechanisms underlying differences in snack timing and the effects of macronutrient-mixed snacks warrant further investigation.

## Figures and Tables

**Table 1:** Nutrition information for the snacks used in the test sessions ^1^.

Snack Group	C Chicken Breast	A Apple	M Macadamia Nut
Weight (g) ^2^	53.0	137.0	9.5
Energy (kcal)	70	70	70
Carbohydrate (g)	3.6	17.1	1.7
Protein (g)	12.9	0	0.8
Fat (g)	0.3	0	6.7

^1^ The nutritional contents of the snacks were obtained from determination experiments (apple) and manufacturers (chicken breast and macadamia nut). ^2^ Water was used to balance the weight differences among snacks.

**Table 2:** The composition and macronutrient and energy contents of the lunch test meals ^1^.

Test Meals	Carbohydrate (g)	Protein (g)	Fat (g)	Energy (kcal)	Detail Content ^2^
Lunch 1 ^3^	96.1	23.3	17.7	637	**uncooked rice 100 g**, roasted sesame dressing 25 mL, **chicken patty 90 g**, cherry tomato 100 g, cucumber 30 g, romaine lettuce 20 g, **sesame oil 4 g**
Lunch 2 ^4^	106.4	25.9	19.8	707	**uncooked rice 112 g**, roasted sesame dressing 25 mL, **chicken patty 101 g**, cherry tomato 100 g, cucumber 30 g, romaine lettuce 20 g, **sesame oil 5.7 g**

^1^ The nutritional contents of the lunch test meals were obtained from China Food Composition Tables and manufacturers. ^2^ The bold parts indicate the differences between the two lunch meals. ^3^ Lunch 1 was used for six snack treatments. ^4^ Lunch 2 was for CON. Bold represents the differences between the two lunch meals.

**Table 3:** Participant baseline characteristics (*n* = 17).

Characteristics	Mean ± SD
Age (years)	21.8 ± 1.4
BMI (kg/m^2^)	21.2 ± 1.7
Waist:hip ratio	0.7 ± 0.0
Fat mass (%)	26.6 ± 2.8
Visceral fat index	2.7 ± 1.0
Basal metabolic rate (BMR) (kcal/day)	1259.4 ± 107.0
Systolic blood pressure (mmHg)	101.6 ± 7.3
Diastolic blood pressure (mmHg)	62.8 ± 5.7

**Table 4:** Postprandial glycemic parameters after snacks and lunch (mean ± SE, *n* = 17).

	Before Lunch (10:30~13:00)	After Lunch (13:00~15:00)				
Test Meals	tAUC_1_ (mmol·min/L)	tAUC_2_ (mmol·min/L)	Max (mmol/L)	LAGE (mmol/L)	CV (%)	J-Index
mid-C	676.4 ± 22.2 ^a^	754.1 ± 22.5 ^cd^	7.5 ± 0.3 ^cd^	3.1 ± 0.2 ^bc^	17.1 ± 1.1 ^bc^	17.4 ± 1.2 ^cd^
pre-C	672.1 ± 20.8 ^a^	736.4 ± 17.3 ^d^	7.4 ± 0.2 ^d^	2.9 ± 0.2 ^cd^	16.1 ± 1.1 ^c^	16.2 ± 0.9 ^d^
mid-A	710.1 ± 21.1 ^a^	802.4 ± 26.2 ^bc^	8.3 ± 0.3 ^abc^	3.8 ± 0.3 ^ab^	20.5 ± 1.3 ^ab^	20.7 ± 1.4 ^bc^
pre-A	700.7 ± 21.5 ^a^	795.8 ± 24.8 ^bc^	7.7 ± 0.2 ^bcd^	2.3 ± 0.2 ^d^	12.1 ± 0.6 ^d^	17.9 ± 1.1 ^cd^
mid-M	681.3 ± 19.6 ^a^	875.7 ± 26.7 ^a^	8.9 ± 0.3 ^a^	4.4 ± 0.3 ^a^	22.5 ± 1.0 ^a^	25.3 ± 1.6 ^a^
pre-M	682.4 ± 18.8 ^a^	834.2 ± 29.5 ^ab^	8.5 ± 0.3 ^ab^	4.0 ± 0.3 ^a^	20.8 ± 1.2 ^a^	22.7 ± 1.8 ^ab^
CON	681.5 ± 26.0 ^a^	840.6 ± 32.6 ^ab^	8.8 ± 0.4 ^a^	4.5 ± 0.3 ^a^	22.6 ± 1.3 ^a^	23.6 ± 2.0 ^ab^

^a,b,c,d^ Different superscript letters denote that mean values within a column are significantly different (*p* < 0.05). The total areas under the glucose curves in two different time periods (10:30~13:00 or 13:00~15:00) are expressed as tAUC_1_ and tAUC_2_, respectively. Mid-C, chicken breast as mid-morning snack; pre-C, chicken breast as preload snack; mid-A, apple as mid-morning snack; pre-A, apple as preload snack; mid-M, macadamia nut as mid-morning snack; pre-M, macadamia nut as preload snack; CON, no snack as control.

**Table 5:** The 24 h glucose parameters for seven snack trail conditions (mean ± SE, *n* = 17).

Test Meals	24 h Mean (mmol/L)	24 h tAUC (mmol·min/L)	24 h Max (mmol/L)	24 h LAGE (mmol/L)	24 h SD	24 h CV (%)
mid-C	4.9 ± 0.1 ^ab^	7018.2 ± 143.1 ^ab^	7.7 ± 0.3 ^c^	4.2 ± 0.3 ^b^	1.0 ± 0.1 ^bc^	20.0 ± 1.4 ^bc^
pre-C	4.8 ± 0.1 ^b^	6998.7 ± 141.1 ^b^	7.7 ± 0.2 ^c^	4.1 ± 0.2 ^b^	1.0 ± 0.0 ^c^	20.1 ± 0.9 ^bc^
mid-A	5.1 ± 0.1 ^a^	7330.1 ± 170.6 ^a^	8.5 ± 0.3 ^ab^	4.7 ± 0.3 ^ab^	1.0 ± 0.1 ^bc^	20.5 ± 0.9 ^abc^
pre-A	5.0 ± 0.1 ^ab^	7241.1 ± 178.7 ^ab^	8.0 ± 0.2 ^bc^	4.2 ± 0.2 ^b^	1.0 ± 0.0 ^c^	19.4 ± 1.1 ^c^
mid-M	5.1 ± 0.1 ^a^	7374.8 ± 146.4 ^a^	9.0 ± 0.3 ^a^	5.2 ± 0.3 ^a^	1.2 ± 0.1 ^a^	23.3 ± 1.3 ^a^
pre-M	5.0 ± 0.1 ^ab^	7255.7 ± 202.4 ^ab^	8.6 ± 0.3 ^ab^	4.8 ± 0.3 ^ab^	1.1 ± 0.1 ^ab^	22.6 ± 1.7 ^ab^
CON	5.0 ± 0.1 ^ab^	7253.3 ± 215.6 ^ab^	8.9 ± 0.3 ^a^	5.1 ± 0.3 ^a^	1.1 ± 0.1 ^ab^	22.4 ± 1.5 ^abc^

^a,b,c^ Different superscript letters denote that mean values within a column are significantly different (*p* < 0.05). Mid-C, chicken breast as mid-morning snack; pre-C, chicken breast as preload snack; mid-A, apple as mid-morning snack; pre-A, apple as preload snack; mid-M, macadamia nut as mid-morning snack; pre-M, macadamia nut as preload snack; CON, no snack as control.

## Data Availability

The data presented in this study are available upon request from the corresponding author. The data are not publicly available due to privacy.
